# EPAS1 expression contributes to maintenance of the primordial follicle pool in the mouse ovary

**DOI:** 10.1038/s41598-024-59382-z

**Published:** 2024-04-16

**Authors:** Jacinta H. Martin, Ilana R. Bernstein, Jess M. Lyons, Ariel R. Brady, Nishani S. Mabotuwana, Simone J. Stanger, Camila Salum De Oliveira, Katerina B. Damyanova, Brett Nixon, Tessa Lord

**Affiliations:** 1 Priority Research Centre for Reproductive Science, Discipline of Biological Sciences, The University of Newcastle, Callaghan, NSW 2308 Australia; 2 Hunter Medical Research Institute, Infertility and Reproduction Program, New Lambton Heights, NSW 2305 Australia

**Keywords:** Oocyte, Folliculogenesis, Primordial follicle, Hypoxia, HIF, EPAS1, Autophagy, Oogenesis, Cell signalling

## Abstract

Oxygen availability can have profound effects on cell fate decisions and survival, in part by regulating expression of hypoxia-inducible factors (HIFs). In the ovary, HIF expression has been characterised in granulosa cells, however, any requirement in oocytes remains relatively undefined. Here we developed a *Hif2a/Epas1* germline-specific knockout mouse line in which females were fertile, however produced 40% fewer pups than controls. No defects in follicle development were detected, and quality of MII oocytes was normal, as per assessments of viability, intracellular reactive oxygen species, and spindle parameters. However, a significant diminishment of the primordial follicle pool was evident in cKO females that was attributed to accelerated follicle loss from postnatal day 6 onwards, potentially via disruption of the autophagy pathway. These data demonstrate the importance of HIF signalling in oocytes, particularly at the primordial follicle stage, and lend to the importance of controlling oxygen tension in the development of in vitro growth and maturation approaches for assisted reproduction.

## Introduction

Dynamic changes in oxygen availability within tissue microenvironments are known to be intertwined with alterations to cellular gene expression, and thus regulation of cell fate decisions, including differentiation and apoptosis^1^. The ovary is one such tissue in which changing concentrations of oxygen are proposed to be interconnected with key developmental processes in the follicle, including regulation of follicular dormancy^2^, ovulation, and corpus lutea formation (reviewed in^3^). Interestingly, despite its reliance on oxidative metabolism^4^, the oocyte develops in a largely avascular environment, separated from the vasculature by the basement membrane, granulosa cells and antral cavity (in the final stages of maturation). Indeed, as the follicle grows, the separation from the vasculature becomes increasingly vast, resulting in a commensurate decline in oxygen tension in follicular fluid^5^ to levels of approximately 1–6%^6,7^. In accordance with habitation in a low-oxygen environment, follicular development is thought to be intricately intertwined with the expression of hypoxia-inducible factors (HIFs)^3^ that play critical roles in adaptation to changing oxygen levels by regulating key downstream processes including cell survival, proliferation, metabolism and angiogenesis^8,9^.

In the HIF signalling pathway, HIF-alpha subunits (HIF1A and HIF2A/EPAS1) are the dynamic regulators of downstream gene expression. The HIF-alpha proteins are stable in hypoxic conditions (< 5% O_2_ or 36 mmHg), facilitating their dimerization with the constitutively expressed HIF-beta subunit ‘ARNT’, and thus binding of hypoxia-response elements (HREs) in promotor regions of target genes^9^. Contrastingly, in conditions > 5% O_2_, HIF-alpha subunits are hydroxylated by prolyl hydroxylases (EGLN1-3), ubiquitinated by the von Hippel-Lindau protein (VHL), and rapidly degraded by the proteasome^10–12^. Although some crossover exists in the suite of known target genes for HIF1A and EPAS1, each does possess the capacity to drive expression of unique cellular processes and signalling pathways^9^. It is worth noting that a third HIF-alpha protein does exist (HIF3A), however it lacks the c-terminal transactivation domain required to dictate downstream expression^13^, so will not be mentioned in further detail here.

Historically, the primary focus of investigation into the role of HIFs in ovarian function has been the granulosa cells. Both HIF1A and EPAS1 have been shown to be expressed in the granulosa cell population, with elevated expression being induced by gonadotropins^14,15^. Accordingly, upregulated HIF-alpha expression in these cells is thought to initiate a cascade of events that include increased vascular endothelial growth factor (VEGF) expression^14^, steroidogenesis^16,17^, cell proliferation^17^, angiogenesis^18^, and formation of the corpus luteum post-ovulation^19^. Conversely, in porcine ovaries, significantly reduced *Hif1a* expression has been found to be associated with follicular atresia in antral follicles^20^.In considering implications for in vitro maturation (IVM) of oocytes, the incubation of cumulus-oocyte complexes in hypoxic culture conditions has been shown to induce HIF-alpha expression in granulosa cells, resulting in upregulation of expression of genes involved in glucose uptake, lipid biosynthesis, mitochondrial function and stress protection^21^. However, similar studies have shown that performing mouse oocyte IVM in artificial low oxygen environments does not culminate in improved fertilisation rates, nor embryo development outcomes^22^, thus highlighting the complexity of the vasculature and oxygen regulation within the ovarian follicle in vivo.

Although HIF signalling pathways are known to be instrumental in directing follicular progression via their expression in the somatic cells in the ovary, the role of HIF expression in the oocyte itself is less well understood. Recently, however, it has been suggested that members of the HIF-alpha family may play an important role in maintenance of the primordial follicle pool^2^. Specifically, using a pluripotent stem cell-based in vitro differentiation culture system, it was demonstrated that hypoxic conditions could drive dormancy in ‘small follicles’ in reconstituted ovaries (equivalent to primordial follicles in the in vivo ovary). By contrast, inhibition of HIF signalling using the dual HIF1A/EPAS1 inhibitor, YC-1, resulted in depletion of the small oocyte pool via precocious oocyte activation^2^. Although these findings suggest that HIF-alpha expression within oocytes plays a role in regulating primordial follicle dormancy, this cannot be unequivocally determined given that somatic cells in the reconstituted ovary would have also been influenced by hypoxia and HIF-inhibitor treatments. Additionally, these studies do not delineate any differential role between HIF1A and EPAS1 within the oocyte itself, although both were demonstrated to be expressed at the mRNA level from embryonic day (E)14.5 and E18.5, respectively^2^.

Given the gap-in-knowledge surrounding the role of HIF-alpha subunits in regulating oocyte development and functionality, we decided to explore this in further detail, with a particular focus on EPAS1. Previous studies have reported that global knockout of *Epas1* results in female infertility, however no assessment of the ovarian phenotype was conducted^23^. In the current study, we generated an *Epas1* germline-specific knockout (*Epas1*^fl/-^) using a *Ddx4*-cre to study the role of EPAS1 in the oocyte. The ovarian phenotype of this conditional knockout (cKO) model was less severe than that of global *Epas1*-knockout animals (likely owing to sustained EPAS1 expression in granulosa cells^15^), however a significant reduction in fertility was still observed. Specifically, *Epas1*-cKO females paired with control males produced 40% fewer pups throughout the breeding period. Assessments of viability, spindle parameters, and levels of cytoplasmic reactive oxygen species (ROS) confirmed that mature oocytes produced from *Epas1*-cKO mice were normal. However, a significantly depleted primordial follicle reserve was identified in females at the onset of sexual maturity (6 weeks of age). In tracing the origins of this primordial follicle depletion back through postnatal development, it was determined that the primordial follicle pool formed normally in the absence of EPAS1 expression, with no evidence of premature oocyte activation. However, from postnatal day (P)6 onwards, a significant acceleration of primordial follicle loss was identified, resulting in a notable reduction in the follicle pool by P14. This increase in follicle atresia appeared to be driven by mechanisms distinct from classical apoptosis, instead being interconnected with a potential disruption to autophagy pathways. Cumulatively, these data support that notion that hypoxia and HIF-alpha signalling molecules are important for the proper development and maintenance of the female germline, in addition to their previously characterised roles in granulosa cell function.

## Results

### Epas1 ablation in the germline causes sub-fertility in female mice

**Figure 1:**
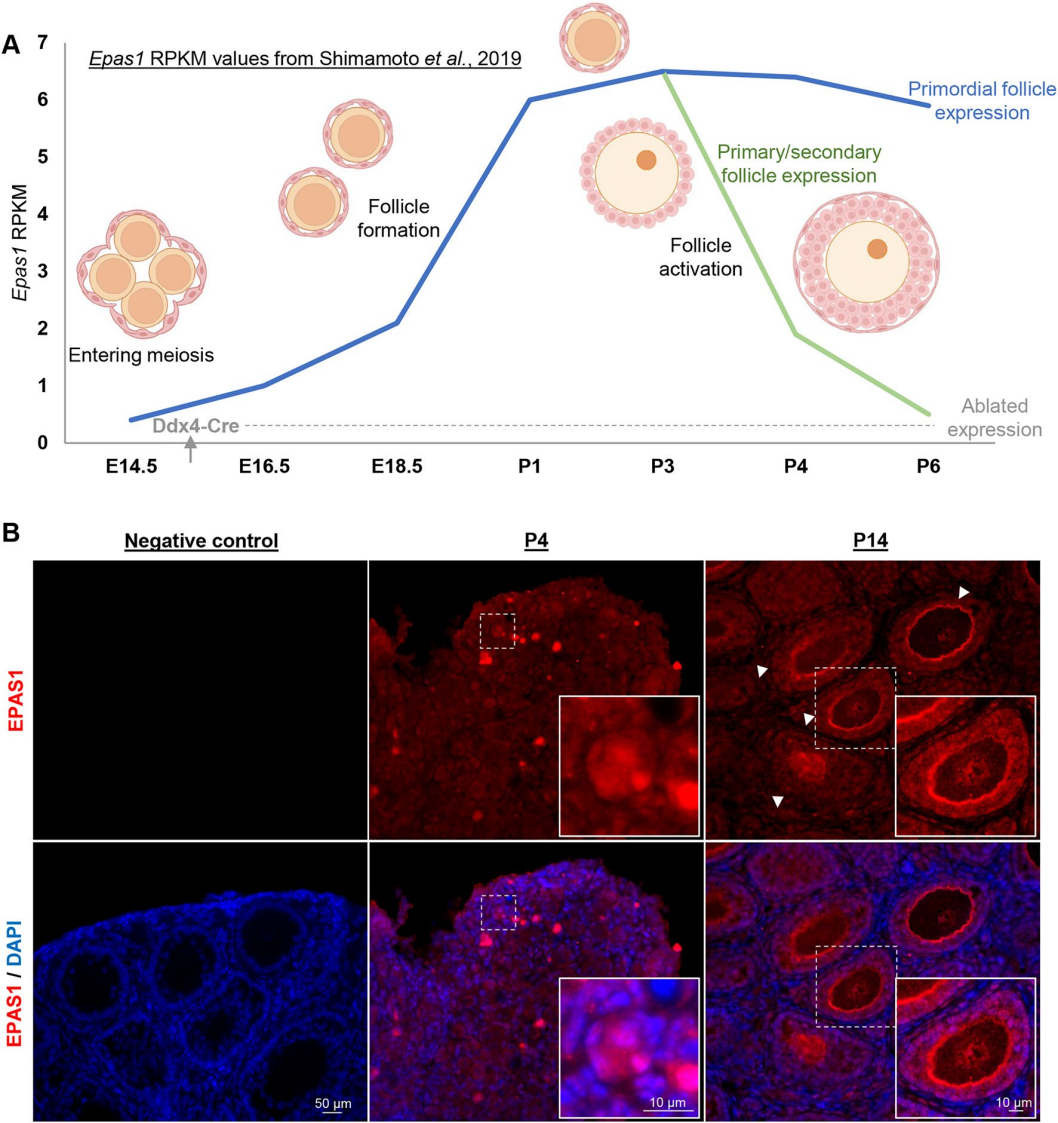
Expression profile of EPAS1 during oocyte development. (**A**) Previously published RNAseq databases (Shimamoto et al., 2019) depict *Epas1* expression in the oocyte from embryonic day (E)16.5, with transcript levels (RPKM values) peaking in primordial follicles at postnatal day (P)3. A decline in *Epas1* expression is observed upon follicle activation. The *Ddx4*-Cre recombinase utilised in this study is active from E15.5, ablating *Epas1* expression prior to the usual period of upregulation. **(B)** Immunofluorescence analyses of EPAS1 expression in the P4 and P14 ovary reflected trends in transcript expression. EPAS1 staining (red) was visible in primordial follicles (see magnified inset image, P4, scale bar = 10 µm), with lower levels of expression being detected in oocytes housed within primary and secondary follicles (white arrowheads and magnified inset image, P14, scale bar = 10 µm). Low levels of EPAS1 expression could also be appreciated in the ovarian cortex (P4) and within granulosa cells surrounding primordial, primary, and secondary follicles (P14). Scale bar = 50 µm.

**Figure 2:**
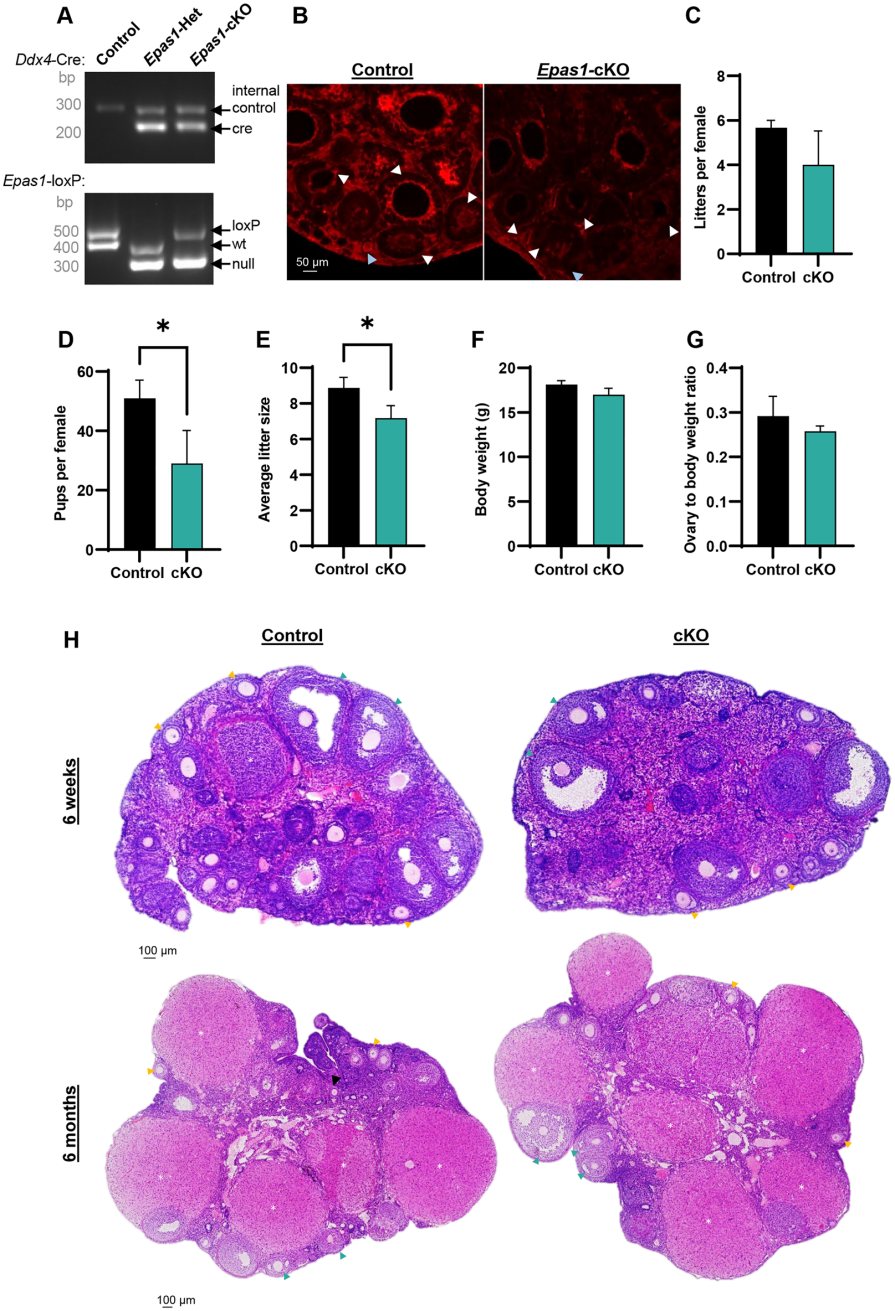
Ablation of EPAS1 in the germline causes female sub-fertility. (**A**) Genotyping analysis reflecting control, heterozygous, and cKO animals (uncropped gel provided in Fig. S1). Ablation of EPAS1 expression in the germline was achieved using *Ddx4*-Cre and *Epas1*-loxP mouse lines. (**B**) Immunofluorescence analyses of EPAS1 expression (red) in control and *Epas1*-cKO ovary sections. In cKO sections, EPAS1 staining was absent from oocytes housed in primordial (blue arrowhead) and primary/secondary (white arrowhead) follicles. Somatic cells retained EPAS1 staining, as expected. Scale bar = 50 µm. (**C**) Control and *Epas1*-cKO females were paired with control males over a breeding period from 6 weeks to 6 months of age. No significant difference was detected in the number of litters produced per female. Histogram shows mean ± S.E.M, n = 3 biological replicates. (**D**) A significant reduction in the number of pups produced per female (throughout the course of the breeding study) was observed in *Epas1*-cKO’s. Histogram shows mean ± S.E.M., n = 3 biological replicates, * indicates statistical significance at *P* < 0.05. (**E**) A significant reduction in average litter size was detected in *Epas1*-cKO females. Histogram shows mean ± S.E.M., n = 3 biological replicates, * indicates statistical significance at *P* < 0.05. **(F)** There was no significant difference in body weight between control and *Epas1*-cKO females. Histogram shows mean ± S.E.M., n = 3 biological replicates. (**G**) There was no significant difference in ovary-to-body weight ratio between control and *Epas1*-cKO females. Histogram shows mean ± S.E.M., n = 3 biological replicates. (**H**) Ovarian histology was found to be overtly normal across all genotypes at both 6 weeks and 6 months of age, as demonstrated by haematoxylin and eosin staining. Scale bar = 50 µm. White asterisks denote examples of Corpus Lutea, black arrowheads primary follicles, yellow arrowheads secondary follicles, green arrowheads tertiary/Graafian follicles.

Although a key role has been established for EPAS1 in facilitating the function of granulosa cells in the hypoxic environment of the developing follicle, any requirement for EPAS1 expression in the oocyte itself remains largely undefined. In order to firstly establish the expression profile of EPAS1 in the oocyte at different stages of development, we collated RNAseq data produced by Shimamoto et al.,^2^, which captured *Epas1* transcript levels in oocytes from E14.5 through to P6 (Fig. 1A). In assessing these data, it can be appreciated that *Epas1* expression in oocytes begins to elevate at the primordial follicle stage from ~ E18.5, and peaks at~ P3. Thereafter, primordial oocytes continued to exhibit high levels of *Epas1* expression out to P6 (further time points not assessed), while oocytes that were recruited into development from P3 onwards (i.e., that had progressed to the primary/secondary stage) experienced a decline in *Epas1* expression. To orthogonally validate these data, we performed immunofluorescence analyses on ovary sections from P4 and P14 mice using anti-EPAS1 antibodies (Fig. 1B). Correlating with transcript abundance, EPAS1 protein expression was evident in primordial oocytes in the P4 ovary. Interestingly, some heterogeneity in EPAS1 expression was evident within the primordial follicle pool, with fluorescence in the nucleus ranging from moderate to bright in intensity. At P14, low levels of EPAS1 expression could be detected within the nucleus of oocytes in primary and secondary follicles. Although a strong signal was also found to be associated within the zona pellucidae of these oocytes, we deemed this to be non-specific based on the persistent zona pellucida cross-reactivity detected in *Epas1-*cKO sections (Fig. 2). Finally, in alignment with previously published literature^15^, significant EPAS1 expression could also be appreciated in somatic cells throughout the ovarian cortex at P4, and within granulosa cells at P14.

Having confirmed EPAS1 expression within the female germ cell, particularly at the primordial follicle stage, we next endeavoured to assess the effects of its ablation on oocyte development and female fertility. We elected to use Cre/loxP recombination technology to knockout *Epas1* in the germ cells specifically, as we have recently reported in the male germline^24^. A *Ddx4*(*Vasa*)-Cre strain was employed^25^, in which Cre is expressed from approximately embryonic day (E)15.5 onwards, achieving recombination in over 95% of oocytes^26^. Thus, in breeding the *Ddx4*-Cre strain with a *Hif2a*(*Epas1*)-loxP mouse strain^27^, *Epas1* deletion is initiated prior to the induction of *Epas1* expression in primordial follicles from E18.5^2^ (Fig. 1A).

To assess the impact of germ cell EPAS1 ablation on female fertility, control (*Epas1*^fl/fl^ or ^fl/+^), and cKO (*Ddx4-Cre*, *Epas1*^fl/-^) (Fig. 2A,B, and S1A,B) female mice were bred with control males from the time of sexual maturity (6 weeks of age), up until 6 months of age (Fig. 2C,D,E). Females were found to be fertile, and there was no significant difference in the number of litters produced over the period of the breeding study (Fig. 2C). However, there was a significant reduction in the number of pups produced per female over the breeding period (Fig. 2D, 51 ± 6.1 pups for control versus 29 ± 5.1 pups for cKO, *P* < 0.05), and in average litter size (Fig. 2E, 8.9 ± 0.6 for control versus 7.2 ± 0.7 for cKO, *P* < 0.05) suggesting that ablation of EPAS1 expression may have caused disruption to the follicle reserve and/or diminished the quality of the oocytes being produced. In breaking down these data to assess the number of pups produced in relation maternal age, a clear reduction in the number of pups produced by cKO females was observed in all age categories (< 3, 3–5, and > 5 months old), however statistical significance was only achieved in the > 5 months old category (Fig. S2A, *P* < 0.05).

To begin to understand the sub-fertility phenotype of *Epas1-*cKO females, we performed a histological comparison of *Epas1-*cKO and control ovaries, using age points that book-ended our breeding study: 6 weeks and 6 months of age. Firstly, it was confirmed that there was no significant difference in body weight between control and *Epas1-*cKO adult female mice (6 week old), in alignment with the germline-specificity of the phenotype (Fig. 2F). In focusing on the ovary, no significant difference was observed in ovary-to-body-weight ratio between genotypes (Fig. 2G), although *Epas1-*cKO ovaries were smaller on average. Haematoxylin and eosin (H&E) staining of ovary sections did not reveal any gross abnormalities in ovarian histology, as expected (Fig. 2H). Assessment of H&E sections also confirmed that folliculogenesis was indeed progressing in the ovary at 6 weeks of age for all genotypes, with an obvious diminishment of follicles being observed by 6 months of age, as per the expected trajectory of reproductive ageing (Fig. 2H).

### Oocyte quality is normal in Epas1-cKO females

**Figure 3:**
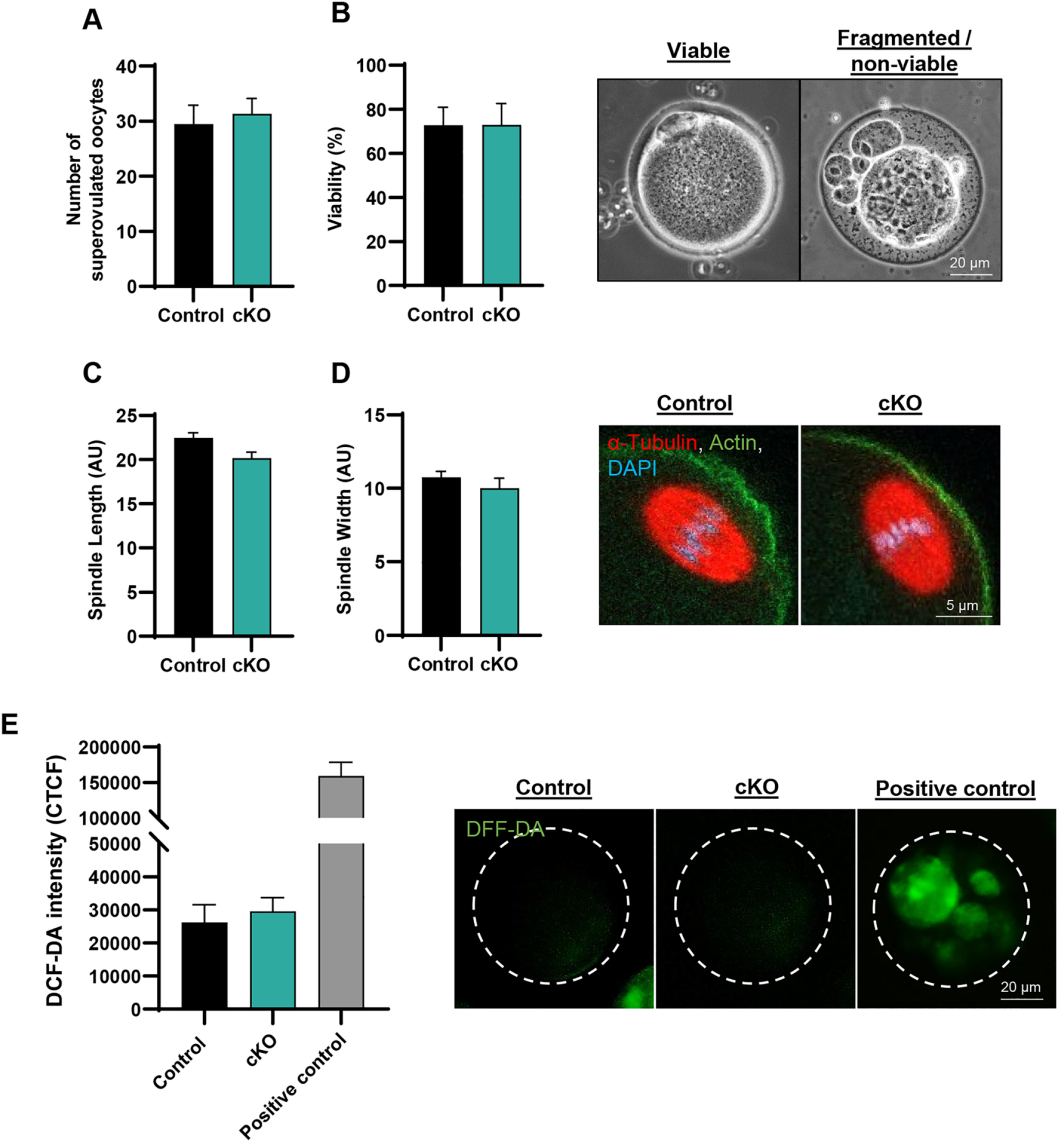
Ablation of EPAS1 expression does not compromise oocyte quality. (**A**,**B**) Superovulation of 4–6 week old control and *Epas1*-cKO females produced comparable numbers of oocytes (A) with no change in levels of oocyte viability (B). Histogram shows mean ± S.E.M., n = 11 and n = 8 biological replicates, respectively. (**C**,**D**) Assessments of length and width of the MII spindle revealed no significant change between control and *Epas1*-cKO oocytes. Graph depicts mean spindle measurements ± S.E.M for oocytes from n = 3 biological replicates. (**E**) Levels of cytoplasmic ROS were not significantly different between control and *Epas1*-cKO oocytes, as determined using the DFF-DA assay. Histogram shows mean ± S.E.M., n = 3 biological replicates.

To explore the underlying cause of sub-fertility in *Epas1-*cKO females, we firstly investigated whether oocyte quality was compromised by assessing a series of parameters in oocytes collected from super-ovulated females at 4–6 weeks of age. Firstly, at this age point, no difference was observed in the number of oocytes collected post-superovulation (Fig. 3A). Similarly, no change was observed in levels of oocyte viability between genotypes (Fig. 3B). Length and width of the MII spindle were also assessed as markers of oocyte quality^28,29^ (Fig. 3C,D respectively), with no significant changes being observed. Finally, given the relationship between EPAS1 expression, mitochondrial function, and oxidative stress^23^, levels of reactive oxygen species (ROS) were assessed using the fluorescent probe DFF-DA^30^. Again, no significant change was observed between control and *Epas1-*cKO oocytes (Fig. 3E). Together, these data suggest that sub-fertility in *Epas1-*cKO females is not related to the production of poor-quality oocytes, at least by the parameters we assessed.

### Precocious diminishment of the primordial follicle pool occurs in the Epas1-cKO ovary

**Figure 4:**
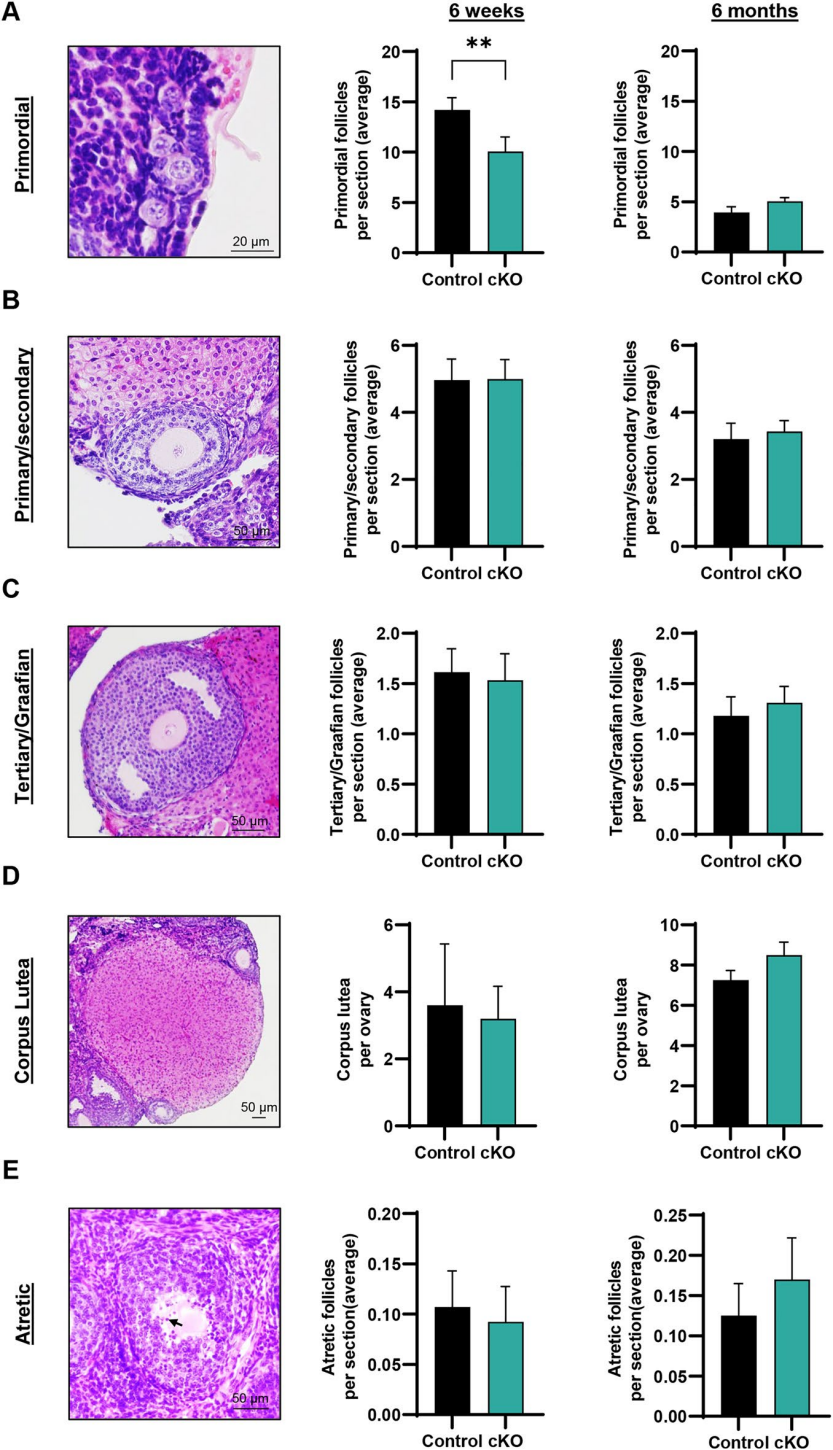
Assessing the effects of ablated EPAS1 expression on follicular reserve and follicle development. (**A**) A significant reduction in the number of primordial follicles was observed in *Epas1*-cKO ovaries when compared to control ovaries at 6 weeks of age. In the 6 month ovary, the primordial follicle pool was equivalently depleted in both the control and cKO (see also Fig S2B, C). Histogram shows mean ± S.E.M., n = 4 biological replicates, ** indicates statistical significance at *P* < 0.01. (**B**) There was no significant difference in the number of primary/secondary follicles when comparing control and *Epas1*-cKO ovaries at either 6 weeks or 6 months of age. Histogram shows mean ± S.E.M., n = 4 biological replicates. (**C**) There was no significant difference in the number of tertiary/Graafian follicles when comparing control and *Epas1*-cKO ovaries at either 6 weeks or 6 months of age. Histogram shows mean ± S.E.M., n = 4 biological replicates. (**D**) The total number of corpus lutea was unchanged when comparing control and *Epas1*-cKO ovaries at 6 weeks and 6 months of age. Histogram shows mean ± S.E.M., n = 4 biological replicates. (**E**) The number of atretic follicles (containing a degenerating oocyte and/or pyknotic granulosa cell nuclei, as denoted by the black arrow), was not found to be statistically different when comparing control and *Epas1*-cKO ovaries at either 6 weeks or 6 months of age. Histogram shows mean ± S.E.M., n = 4 biological replicates.

Next, we elected to perform a detailed quantitative analysis of follicle development (primordial, primary/secondary, and tertiary/Graafian: representative images provided in Fig. 4A–C), again focusing on 6 week and 6 month time points. Interestingly, a significant reduction in the average number of primordial follicles per ovarian section was detected in 6 week old *Epas1-*cKO females when compared to control females (Fig. 4A, 14 ± 1.2 for control versus 10 ± 1.5 for cKO, *P* < 0.01). In extrapolating these counts to reflect the entire primordial follicle pool in the ovary (using serial sectioning methodologies described previously^31^), a~ 30% reduction the total number of primordial follicles was observed (*P* < 0.05, Fig. S2B). This reduction in primordial follicle number did not extrapolate into a reduction in the number of primary/secondary or tertiary/Graafian follicles observed in the 6-week ovary (Fig. 4B,C), suggesting that the reduced primordial follicle pool that remained did not experience any impairments in recruitment/activation. Additionally, the total number of corpus lutea per ovary was not statistically different between genotypes (Fig. 4D), aligning with superovulation data in Fig. 3A.

In assessing ovaries from reproductively ‘aged’ (6-month-old) females, the primordial follicle pool was found to be equally diminished in both the control and *Epas1-*cKO, such that there were no longer significant differences between genotypes (Fig. 4A, S2C). Accordingly, no differences were observed between genotypes in the primary/secondary or tertiary/Graafian stages in the 6-month ovary (Fig. 4B,C), nor in the number of corpus lutea (Fig. 4D).

As an additional parameter for assessment of ovarian histology, we also quantified the number of developing follicles that were atretic, as identified by the presence of a degenerating oocyte and/or pyknotic granulosa cell nuclei (arrow, representative image in Fig. 4E). No significant differences in atretic follicle counts were identified at either 6 weeks or 6 months of age (Fig. 4E).

### Epas1 ablation in the germline does not cause premature follicle activation, however, does cause accelerated primordial follicle loss in the postnatal ovary

**Figure 5:**
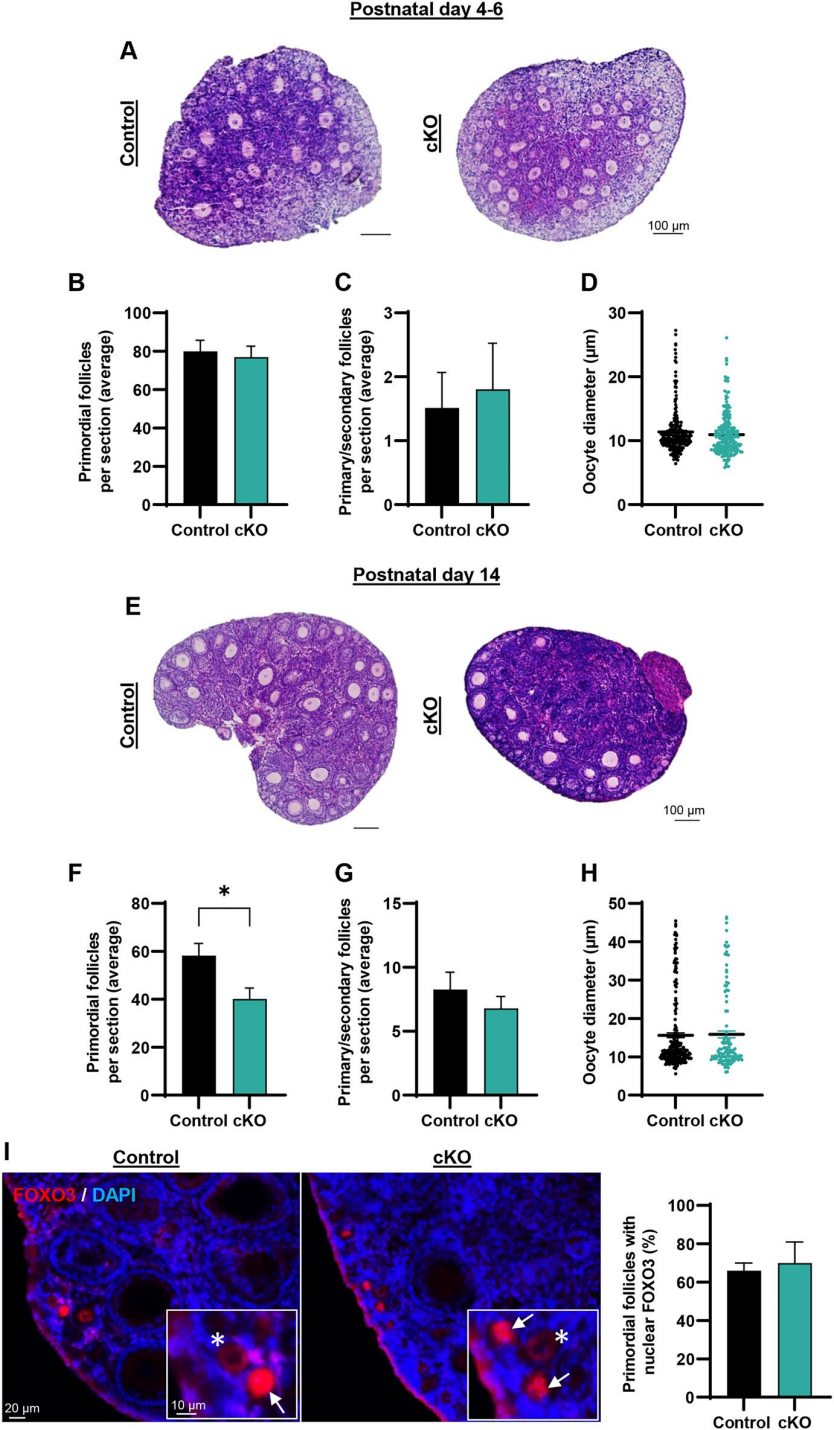
Assessing primordial follicle formation and activation in the postnatal ovary of Epas1-cKO females. (**A**,**B**) The average number of primordial follicles per ovary section was equivalent in control and *Epas1*-cKO ovaries of postnatal day 4–6 females. (**A**) Representative images of ovary sections stained with haematoxylin and eosin, (**B**) histogram depicting the number of primordial follicles per ovary. Histogram shows mean ± S.E.M., n = 6 biological replicates. (**C**) There was no significant difference in the number of primary/secondary follicles in control and *Epas1*-cKO postnatal day 4–6 ovaries. Histogram shows mean ± S.E.M., n = 6 biological replicates. (**D**) A comparison of oocyte diameter within developing follicles, comparing control and *Epas1*-cKO postnatal day 4–6 ovaries, revealed no significant differences. Graph depicts individual follicle measurements and mean, for n = 4 biological replicates. (**E**,**F**) A significant decline in primordial follicle numbers was detected in *Epas1*-cKO ovaries at postnatal day 14, as compared to controls. (E) Representative images of ovary sections stained with haematoxylin and eosin. (F) Histogram depicting the number of primordial follicles per ovary. Histogram shows mean ± S.E.M., n = 4 biological replicates. * indicates statistical significance at *P* < 0.05. (**G**) There was no significant difference in the number of primary/secondary follicles in control and *Epas1*-cKO postnatal day 14 ovaries. Histogram shows mean ± S.E.M., n = 4 biological replicates. (**H**) A comparison of oocyte diameter within developing follicles, comparing control and *Epas1*-cKO postnatal day 14 ovaries, revealed no significant differences. Graph depicts individual follicle measurements and mean, for n = 4 biological replicates. (**I**) FOXO3 staining (red) overlaid with the nuclear stain DAPI (blue) in postnatal day 14 ovaries from control and *Epas1*-cKO mice. No significant change in the percentage of oocytes with nuclear staining (‘dormant state’, arrow in inset) versus cytoplasmic staining (‘activated state’, asterisk in inset) could be identified. Histogram shows mean ± S.E.M., n = 3 biological replicates. Scale bar = 20 µm (10 µm in inset).

To better understand the underlying basis for depletion of the primordial follicle pool observed in 6 week old *Epas1-*cKO females, we assessed the ovarian phenotype at earlier stages in development. Given that previous research suggested an association between hypoxia and primordial follicle dormancy in in vitro reconstituted ovaries (that resemble the early postnatal ovary (pre-P6)^2^), we began by assessing control and *Epas1-*cKO ovaries at P4-P6. In evaluating H&E sections (Fig. 5A) and performing follicle counts, no significant difference in the number of primordial follicles (Fig. 5B, S2D), nor in the number of primary/secondary follicles (Fig. 5C) could be observed. Accordingly, when measuring the average follicle diameter at this age, no significant difference was found (Fig. 5D). These results suggest that (1) establishment of the primordial follicle pool is not impaired in *Epas1-*cKO mice, (2) the depletion of primordial follicles in these mice occurs at a time point beyond P6, and (3) germline ablation of EPAS1 does not instigate precocious activation of oocytes at this time point.

To extend this assessment further into ovarian development, we performed an equivalent suite of experiments on ovaries at P14 (Fig. 5E–I). P14 was selected given that this is the timepoint in which a precocious follicle activation phenotype was observed in *Foxo3a-/-* female mice^32^, with a potential association between hypoxia, HIFs, and FOXO3A expression being previously reported^2^. Interestingly, at P14 we observed the emergence of the primordial follicle depletion phenotype that was identified in the 6 week ovary, here manifesting as a ~ 25% reduction in the primordial follicle pool (Fig. 5E,F *P* < 0.05, and S2E, *P* < 0.05). If these primordial follicles were being lost to precocious activation in the *Epas1*-cKO, we would expect to see an increase in primary/secondary follicle numbers, as reported in Castrillon et al.,^32^, however no significant differences in primary/secondary follicles were observed (Fig. 5G), nor was any increase in average follicle diameter (Fig. 5H). Additionally, no changes in the percentage of primordial follicles with nuclear FOXO3A staining (“dormant” state), as opposed to cytoplasmic staining (“activated” state)^32^ could be observed between genotypes, with ~ 60% of primordial follicles from both genotypes exhibiting nuclear expression (Fig. 5I) in line with previous reports for this age range^33^.

**Figure 6:**
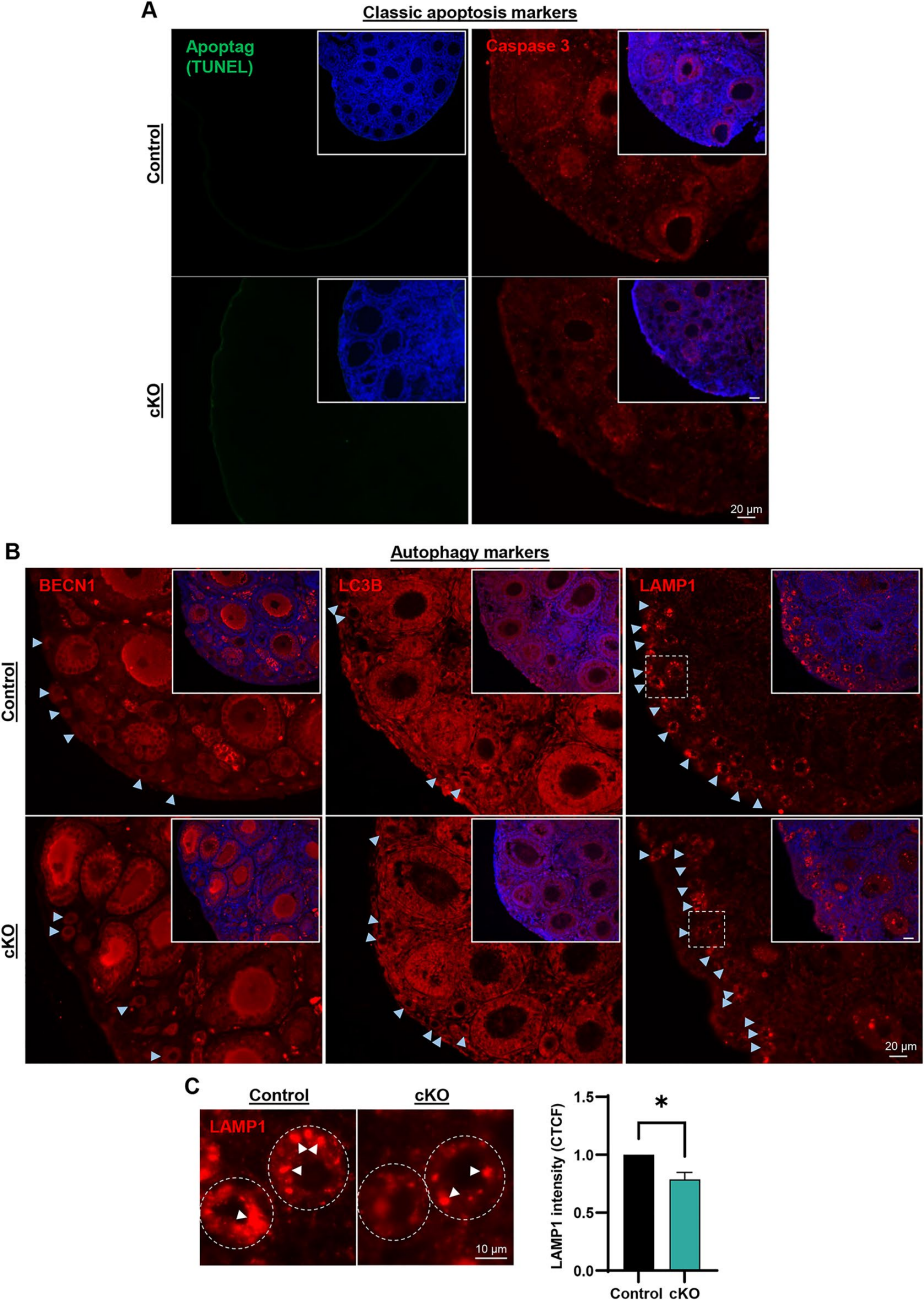
Exploring the mechanism behind primordial follicle loss in the *Epas1*-cKO ovary. (**A**) The acceleration of primordial follicle atresia in the *Epas1*-cKO P14 ovary was not accompanied by increased detection of classical apoptotic markers: Apoptag/TUNEL (left) and cleaved Caspase 3 (right). Positive and negative control images are provided in Fig. S3A, B. Scale bar = 20 µm. (**B**) Assessment of expression of the autophagy markers BECN1, LC3B, and LAMP1 in control and *Epas1*-cKO P14 ovaries, with a specific focus on primordial follicles (blue arrowheads). Negative control images are provided in Fig. S3C. Dashed boxes are magnified images in (C). Scale bar = 20 µm. (**C**) Primordial oocytes from *Epas1*-cKO ovaries had fewer large LAMP1 expressing foci within their cytoplasm (white arrowheads, scale bar = 10 µm), culminating in a significant reduction in Corrected Total Cell Fluorescence (CTCF) values for LAMP1 expression across n = 3 biological replicates. Histogram shows mean ± S.E.M., * indicates statistical significance at *P* < 0.05.

Cumulatively, these data suggested that ablation of EPAS1 expression in the germline was instigating an accelerated loss of primordial follicles to atresia in the pre-pubertal ovary, as opposed to precocious activation. Interestingly, however, an assessment of apoptotic markers (cleaved caspase 3 and DNA fragmentation (TUNEL assay)) revealed no appreciable differences between control and *Epas1*-cKO ovaries at P14 (Fig. 6A and S3A, B), aligning with previous studies that report that primordial follicle atresia in the pre-pubertal ovary occurs via mechanisms that are distinct from classical apoptotic pathways^34–37^. Additionally, we found no evidence of structures resembling ‘empty’ degenerating follicles, such as those previously reported in other mutant mouse lines with a compromised follicle reserve^38^. We did, however, uncover evidence for disrupted autophagy pathways that have been linked with maintenance of the primordial follicle pool in previous studies^39–41^, and that are known to be driven by HIF pathways to promote cell survival in other cell types^42^. Specifically, we compared expression of the early, mid, and late autophagy markers Beclin-1 (BECN1), Autophagy marker Light Chain 3B (LC3B), and Lysosomal Associated Membrane Protein 1 (LAMP1), respectively, in control and *Epas1*-cKO P14 ovaries. Expression of all three markers was detected within the cytoplasm of primordial oocytes (Fig. 6B, blue arrowheads). BECN1 was also expressed in primary/secondary stage oocytes, and within granulosa cells surrounding primary follicles, while LC3B exhibited expression in granulosa cells of follicles at all stages of development. LAMP1 expression, however, was specific for primordial oocytes. No differences in the expression of BECN1 and LC3B could be appreciated between the control and *Epas1*-cKO ovary. However, primordial oocytes in the *Epas1*-cKO did appear to contain a reduced number of large, brightly stained LAMP1 foci in their cytoplasm (Fig. 6B,C white arrowheads) when compared to control oocytes, with pixel intensity analysis revealing a significant reduction in LAMP1 fluorescence in the cKO (Fig. 6C, *P* < 0.05) that could potentially reflect disruption to the later stages of autophagy in these cells.

**Figure 7:**
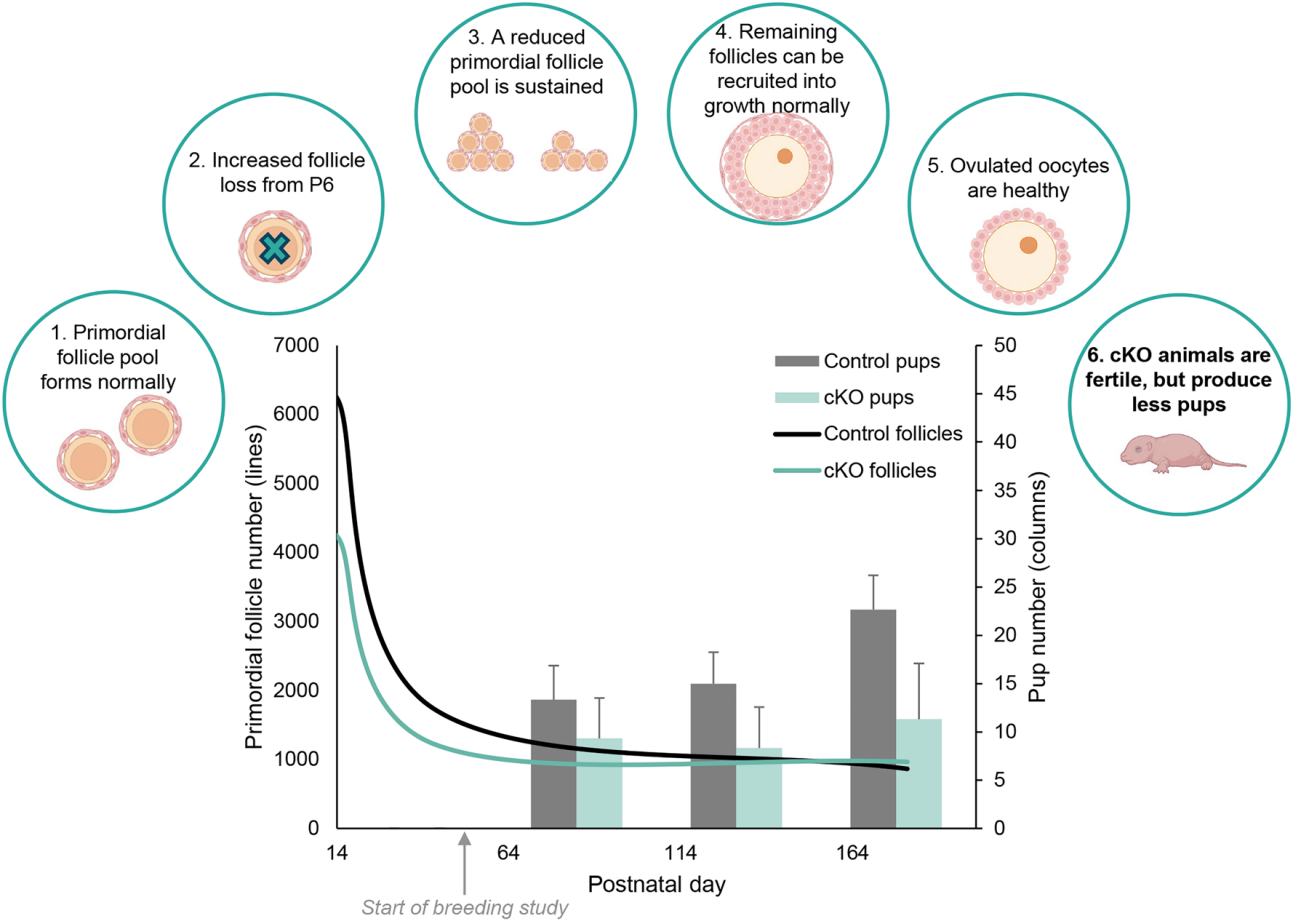
Summary. Mice with germline ablation of *Epas1* (*Epas1*-cKO) show normal formation of the primordial follicle pool, however experience accelerated follicle loss from P6 onwards, potentially due dysfunction of the autophagy pathway. A reduced primordial follicle pool is sustained until sexual maturity, and these follicles can be recruited normally and support the development of healthy oocytes. Regardless, the diminished follicle pool in *Epas1*-cKO females culminates in sub-fertility, with cKO females producing significantly less pups than their control counterparts. Left axis: predicted size of primordial follicle pool (summary of data from Fig. 4, 5 and S1). Right axis: average number of pups produced within different time frames of the breeding study: 0–50, 50–100, and 100–150 + days post-initiation of breeding (summary of data presented in Fig. 2C, D, and S2A).

In conclusion, experiments described in this manuscript demonstrate that EPAS1 expression in the female germline is important for normal female fertility. In the absence of EPAS1 expression, accelerated loss of the primordial follicle pool ensues from P6 onwards (Fig. 7), potentially as a consequence of dysregulated autophagy pathways, resulting in a reduced primordial follicle pool in the adult ovary. Accordingly, *Epas1-*cKO females are fertile, but produce fewer pups than their control counterparts throughout their reproductive life span, with the consequences of a reduced primordial follicle pool becoming particularly evident by 5 months of age, where fecundity is halved (Fig. 7).

## Discussion

The importance of hypoxia and hypoxia inducible transcription factors in regulating ovarian function has long been appreciated^3^, however the intricacies surrounding HIF1A versus EPAS1 signalling, and the comparative roles of these transcription factors in regulating granulosa cell versus germ cell function, remain relatively unexplored. Here, we have provided the first characterisation of the role of EPAS1 within the oocyte specifically. Female mice with germline knockout of *Epas1* experienced sub-fertility, producing fewer pups than their control littermates. Oocyte quality within these mice was normal, however a significant diminishment in the primordial follicle pool was evident by P14, which was found to be intertwined with elevated levels of follicle loss, potentially due to disruption of the autophagy pathway (summarised in Fig. 7). Such findings build our appreciation of the complexity of oxygen tension requirements for oocyte growth and maturation in vitro, and also to the potential reproductive consequences of HIF-modulating drugs that are used in the treatment of some human diseases (discussed below).

In assessing the protein profile of EPAS1 in the mouse ovary, EPAS1 expression was primarily evident in primordial oocytes, with heterogeneous expression evident throughout the primordial follicle pool. Low levels of EPAS1 expression were also identified in the nucleus of the oocyte from the primary stage onwards (Fig. 1B). This is perhaps surprising when considering that the oocyte is thought to be subjected to elevated levels of hypoxia as the follicle grows, commensurate with the increasing distance between the oocyte and vasculature^5^. However, this finding does align with previously published RNAseq data^2^, which depict elevated levels of *Epas1* transcript in immature female germ cells (peaking at ~ P3), with a precipitous decline in expression accompanying the primary/secondary stages of follicle development (Fig. 1A). Certainly, the phenotype observed in germline-specific knockout mice in this study corresponds with EPAS1 having a particularly important role at the primordial follicle stage (to be elaborated upon shortly), with no obvious role identified in regulating the quality or survival of oocytes in the growth stage of folliculogenesis or post-ovulation. Thus, although loss of EPAS1 expression is known to be interconnected with dysregulated mitochondrial function and metabolism, and increased levels of ROS in other tissue types^23^, we did not uncover any evidence to suggest that this is the case within the mature oocyte (Fig. 3E). It remains to be determined whether HIF1A plays a similar role within the female germ cell, however it is worth noting that HIF1A protein has been detected in oocytes at the later stages of folliculogenesis^43^. Further, it is well defined that HIF1A and EPAS1 do regulate different downstream genes and pathways, despite some crossover in their gene targets^9^. Thus, it is possible that HIF1A has a functional role later in oocyte development, while the role of EPAS1 is restricted to the primordial follicle stage.

By far the most prominent aspect of the phenotype in *Epas1* germline knockout females was the diminishment of the primordial follicle pool. Indeed, although primordial follicles formed normally in these mice with equivalent numbers being present at P4-6, by P14 the number of primordial follicles had reduced by 25% in the *Epas1*-cKO as compared to the control (Fig. 5F). The modest nature of this phenotype may be related to the heterogeneity in EPAS1 protein expression observed within the primordial follicle pool, however the significance of this heterogeneity warrants additional investigation in future studies. Regardless, the phenotype observed in the *Epas1*-cKO is, in part, reminiscent of that described in in vitro recombinant ovaries that had been exposed to normoxia (rather than hypoxia), or to the dual HIF1A/EPAS1 inhibitor YC-1^2^, however with some important differences. In recombinant ovaries cultured in hypoxia, the ‘small oocyte’ pool (equivalent to primordial follicles in the in vivo ovary) was established and sustained in a semi-dormant state, while in normoxia (or following YC-1 treatment), small oocytes were rapidly depleted by way of premature recruitment into development^2^. Contrastingly, in the *Epas1*-cKO mouse, no premature activation of primordial follicles was identified, with diminishment of the primordial follicle pool instead being attributed to accelerated follicle loss (Fig. 5, 6 and Fig. S3).

In considering the difference in phenotypes between our in vivo analysis of the *Epas1*-cKO ovary and that of reconstituted ovaries treated with YC-1^2^, we again revisit the notion that different downstream signalling pathways are regulated by EPAS1 and HIF1A in the oocyte. Shimamoto et al.^2^ characterised FOXO3A as one downstream effector of hypoxia / HIF signalling that was directly regulated to maintain follicle dormancy in reconstituted ovaries. Indeed, FOXO3A is known to be key for primordial follicle maintenance, with the FOXO3A knockout mouse line experiencing overactivation of immature follicles by P14, resulting in infertility by 15 weeks of age^32^. In our study, we did not find evidence in *Epas1*-cKO ovaries that suggested a defect in FOXO3A signalling. In this vein, there was no elevation in the number of primary/secondary follicles observed at P14, no changes in follicle diameter, and no changes in FOXO3A localisation between genotypes (Fig. 5). Again, we can postulate that suppression of activation through oocyte-intrinsic FOXO3A signalling observed in reconstituted ovaries in hypoxia^2^ is likely to be an effect of HIF1A signalling, rather than EPAS1 signalling, particularly given that HIF1A does appear to be the more prominent regulator of FOXO3A, based on siRNA knockdown studies^44^. Also worth considering is the fact that hypoxia and YC-1 exposures in reconstituted ovaries can potentially influence surrounding somatic cells, which could disrupt exogenous signals regulating follicle dormancy that would not be present in our germline-specific knockout model. Regardless, our data support a model in which the primary mechanism of primordial follicle depletion caused by EPAS1 ablation in the oocyte is accelerated follicle loss, rather that premature activation (Fig. 5, 6).

Of interest in this study was the significant reduction in primordial follicle numbers in the *Epas1*-cKO ovary without an accompanying increase in expression of classical apoptotic markers (active Caspase 3 and TUNEL) (Fig. 6). This finding contributes to a growing body of literature that suggests that primordial follicle atresia in the pre-pubertal ovary is governed by mechanisms that are distinct from classical apoptosis pathways^34–37^. Given that hypoxia and HIF signalling are known to drive the activity of autophagy pathways that promote cell survival in other cell types^42^, and recent literature that suggests that the activity of these autophagy pathways is required to prevent accelerated loss of the primordial follicle pool^39–41^, we were particularly interested in uncovering any disruption to these pathways in *Epas1*-cKO ovaries. Indeed, in the case of LAMP1, a late-stage autophagy marker that exists within the lysosomal membrane, we did detect modest changes in expression. Specifically, a reduced number of intensely stained foci were detected in the primordial oocyte cytoplasm, potentially reflecting alterations in lysosome availability for fusion with autophagosomes, and thus reduced protein turnover within the cell, as has been proposed to occur during reproductive ageing of mature oocytes^45^. Despite this, no discernible differences could be determined in expression in the early- and late- autophagy markers BECN1 and LC3B. Thus, whether the findings reported here translate into functional changes in autophagy capacity and survival of primordial oocytes should be the focus of future investigations. Regardless, the determination of expression profiles for three recognised autophagy markers in the P14 ovary (BECN1, LC3B, LAMP1), contributes to a growing body of literature that suggests a key role for autophagy within the primordial oocyte, that perhaps shifts to the granulosa cells upon recruitment of follicles into development, as proposed in Zhou et al.^40^.

In overlaying findings from this manuscript onto previously published literature, we can begin to form a more holistic picture of the role of hypoxia and HIFs in regulating ovarian function. Broadly, data produced here and elsewhere^2^ suggest that oocyte expression of HIFs is particularly important for maintenance of the primordial follicle pool, although further investigation is required to consolidate the role of HIF1A. Contrastingly, HIF expression in granulosa cells (seemingly both HIF1A and EPAS1) is important in the latter stages of folliculogenesis for unencumbered ovulation and formation of the corpus luteum^15^. This, and the total infertility phenotype that ensues in global *Epas1* knockout mouse lines^23^, underscores the importance of controlled oxygen tension in developing in vitro growth (IVG) and IVM technologies for clinical practice, particularly for women and girls who are seeking to safeguard their future fertility in response to a cancer diagnosis^46^ or for those at risk of ovarian hyperstimulation syndrome^47^. In considering the utility of IVM, significant improvements have been made in these technologies such that live-birth rates are now up to 50% per embryo transfer^48^. However, the blastocyst formation rate and cumulative live birth rate resulting from IVM remains below that achieved using standard in vitro fertilisation (IVF) approaches^48–51^. Regarding IVG of primordial follicles captured in cryopreserved ovarian tissue, this approach remains considerably more challenging and is not yet offered in a clinical setting^46^. Certainly, promising results have been produced using IVG in mouse models^52,53^ and human tissue^54,55^, however the potential implications for offspring health remain a concern. In optimising these in vitro assisted reproduction techniques to maximise the chance of a successful pregnancy, replication of the physiological conditions of the ovary in an in vitro setting is an important component. However, mimicking in vivo conditions in an in vitro environment is undeniably complex, as can be appreciated by proteomic profiling of human oocytes that had gone through IVM in hypoxia (5% O_2_), which continued to exhibit differentially expressed proteins when compared to in vivo matured oocytes, as well as a high level of inter-group heterogeneity^56^. Beyond the applications for assisted reproduction technologies, the phenotype described in *Epas1*-cKO females in this study also highlights the need for consideration when administering EPAS1 inhibitors (e.g. Belzutifan) for the treatment of rare diseases (such as polycythemia and paraganglioma), and von Hippel–Lindau (VHL) associated tumours^57^. This is particularly the case for young female patients, (as in Kamihara et al.,^58^) who may wish to be informed on the potential effects to their future fertility.

In conclusion, here, we have provided the first evidence that EPAS1 expression in the female germ cell is important for survival and maintenance of the primordial follicle pool in the mouse ovary. Coinciding with this, we have demonstrated that conditional knockout of *Epas1* within the oocyte causes sub-fertility, with *Epas1*-cKO female mice producing fewer pups over the breeding period than their control counterparts. Understanding of the role of oxygen tension and hypoxia-inducible signalling pathways in ovarian function is an important gateway to improving in vitro manipulation techniques for female gametes, but also for broadly understanding the molecular mechanisms underlying fertility and sub-fertility.

## Materials and methods

### Animals

All methods were performed in accordance with the relevant guidelines and regulations. Procedures involving animal use were approved by the University of Newcastle Animal Care and Ethics Committee (ACEC, approval number A2019-907) and were carried out in accordance with the Australian code for the care and use of animals for scientific purposes and ARRIVE guidelines. To ablate EPAS1 expression in oocytes, previously established *Ddx4(Vasa)*-Cre^25^ and *Epas1*-loxP^27^ mouse lines were obtained from the Jackson Laboratory (Bar Harbor, ME, USA; stock numbers 006954 and 008,407, respectively). *Ddx4*-Cre, *Epas1*^-/+^ males were bred with *Epas1*^fl/fl^ females to generate conditional knockout animals (*Ddx4*-Cre, *Epas1*^fl/-^) used in this study. Primer sequences used for genotyping are provided in Table S1.

### Follicle counts and measurements

Follicle counts were performed using a serial sectioning and direct count approach that has been described and validated previously^31^. Briefly, ovaries were fixed in Bouin’s solution (Sigma Aldrich, St Louis, MO, USA) for 24 h, subjected to a series of ethanol washes, and embedded in paraffin blocks. Ovaries were then serial sectioned through the entire volume of the tissue, creating sections of 5 µm thickness that were subsequently placed on microscope slides. To visualise the different stages of follicle development, Haematoxylin and Eosin staining and direct counts were performed on every ninth section. Follicles were categorised as primordial, primary/secondary, tertiary/Graafian, or atretic, as per the representative images provided in Fig. 4. The total number of corpus lutea per ovary was also counted, with all sections being assessed to ensure that each corpus luteum was only counted once. Follicles were only included in raw data counts if the nucleus was visible. To generate a numerical value to reflect the entire primordial follicle pool in the ovary, raw counts were multiplied by 9 to account for unassessed ovarian sections (as described previously^31^). In experiments where follicle measurements were conducted, the measurement function in Image J (National Institute of Health) was utilised to assess oocyte diameter.

### Immunofluorescence analysis of ovary sections

Immunofluorescence analysis was performed on Bouin’s fixed ovary sections, as described previously^59^. Sections were de-paraffinised and rehydrated via a series of xylene and ethanol washes (100%, 75% and 0% ethanol in H_2_O), respectively. Antigen retrieval was conducted via a 10 min incubation in boiling sodium citrate buffer (10 mM, pH 6: for anti-EPAS1, anti-Caspase 3, anti-BECN1, anti-LC3B, and anti-LAMP1 antibodies) or Tris buffer (10 mM, pH 10: for anti-FOXO3 antibody). Ovary sections were blocked from non-specific antibody interactions using 3% bovine serum albumin (BSA; Sigma Aldrich) and 10% goat serum (Sigma Aldrich), diluted in phosphate buffered saline (PBS; Sigma Aldrich). Primary antibody incubation (anti-EPAS1, Novus Biologicals #NB100-122; anti-FOXO3A, Cell Signalling Technology #2497; anti-Caspase 3, Abcam #ab13847; anti-BECN1, Novus Biologicals #NB500-249; anti-LC3B, Abcam #ab48394; or anti-LAMP1, Abcam #ab24170) was conducted overnight at 4 °C using a 1/100 (FOXO3A, Caspase 3) or 1/200 (BECN1, LC3B, LAMP1) dilution in 1% BSA/PBS. A negative control was also conducted in which primary antibody was omitted. Following a series of washes in PBS, sections were then incubated in a 1/200 secondary antibody solution (Alexa Fluor anti-rabbit 594; Thermo Fisher Scientific, Waltham, MA, USA) for 1 h at room temperature. Finally, sections were incubated with DAPI (1/1000, Sigma Aldrich) for 5 min, before being washed and mounted on microscope slides in Mowiol containing 1,4-diazabicyclo[2.2.2]octane (DABCO) (Sigma Aldrich). Immunofluorescent images were captured using a Zeiss Axio A.2 fluorescence microscope (Carl Zeiss Micro Imaging GmbH, Jena, Thuringia, Germany). Corrected Total Cell Fluorescence (CTCF) values were calculated using Image J.

As an additional measure to identify apoptotic follicles in ovary sections, an ApopTag^®^ Fluorescein In Situ Apoptosis Detection Kit was used (Sigma Aldrich). These experiments were performed as above, with the following alterations. Following tissue rehydration, sections were treated with 20 µg/mL proteinase K for 15 min at room temperature, and positive control sections were subsequently treated with DNase for 10 min. Tdt enzymes and anti-digoxigenin conjugate were then sequentially added to sections, as per the manufacturer’s instructions.

### Superovulation and oocyte collection

Superovulation was conducted as described previously^59,60^. 4–6 week old females were subjected to an injection regimen of equine Chorionic Gonadotropin (eCG) (Intervet, Sydney, Australia), followed by human Chorionic Gonadotropin (hCG) (Intervet), 48 h later. Oocytes were harvested at 15 h post-hCG injection. Briefly, cumulus-oocyte-complexes were collected from the oviductal ampullae and placed in a 300 µg/ml hyaluronidase (Sigma Aldrich) solution for 2–5 min at 37 °C to liberate oocytes from the surrounding cumulus cells. Oocytes were then washed 3 times in M2 medium (Sigma Aldrich). Metaphase II (MII) stage oocytes were identified by the presence of a single polar body and absence of a germinal vesicle.

### Immunofluorescence analysis to assess oocyte spindle integrity

To facilitate morphometric assessment of the meiotic spindle, oocytes were fixed in a 3.7% paraformaldehyde solution for 45 min^29,59^, and permeabilised in 0.25% Triton X-100 (Sigma Aldrich) in PBS for 10 min at room temperature. Prior to antibody staining, oocytes were placed in a blocking solution of 3% BSA/PBS for 1 h at 37 °C. Primary antibody incubation was conducted overnight in anti-α-tubulin (1:400, Thermo Fisher Scientific, #A11126). Oocytes were then washed in 1% BSA/PBS prior to incubation with an anti-mouse Alexa Fluor 594 conjugated secondary antibody (Thermo Fisher Scientific) diluted 1:1000 in 1% BSA/PBS at 37 °C for 1 h. Dual labelling was achieved the by sequential incubation in a fluorescent phalloidin conjugate (which labels polymeric actin) for 20 min at 37 °C^61^. All cell preparations were counterstained with the nuclear marker DAPI and mounted onto Menzel Glӓser microscope slides (Thermo Fisher Scientific) in antifade reagent (Prolong Gold Antifade, Thermo Fisher Scientific). The Zeiss LSM 900 confocal Z stacking function was utilised to record the dimensions of the entire spindle from pole to pole. Finally, the images collected were again imputed into Image J and analysed using a custom spindle analysis tool/macro designed by Dr. S. Lane, thus facilitating calculation of spindle size and length^62^.

### Carboxy-DFFDA assay

To assess levels of cytoplasmic ROS in oocytes, a 5′-carboxy-2′,7′-difluorodihydrofluorescein diacetate (carboxy-DFFDA; Molecular Probes) assay was used, as described previously^30^. Specifically, oocytes were incubated in 10 µM carboxy-DFFDA for 15 min, followed by 3 washes in PBS containing 3 mg/ml polyvinylpyrrolidone. Oocytes were imaged immediately on a Zeiss Axio A.2 fluorescence microscope and CTCF values were calculated using Image J.

### Data analyses

All experiments were conducted a minimum of three times, on independent biological replicates (i.e. from different animals). Data are presented as Mean ± S.E.M. Statistical differences were established using the ANOVA, t-test, or Mann–Whitney function in GraphPad Prism 9 software. A value of *P* < 0.05 was considered to be statistically significant.

### Supplementary Information

Supplementary Information.

## Data Availability

The datasets used and/or analysed during the current study available from the corresponding author on reasonable request.
