# Supplementary material for: EPAS1 expression contributes to maintenance of the primordial follicle pool in the mouse ovary
Source: Sci Rep. 2024 Apr 16;14:8770. doi: 10.1038/s41598-024-59382-z (PMC11021563; doi:10.1038/s41598-024-59382-z)
Supplement: Supplementary file 1 — Supplementary Information. [file 41598_2024_59382_MOESM1_ESM.pdf]

SUPPLEMENTAL FIGURE 1

A

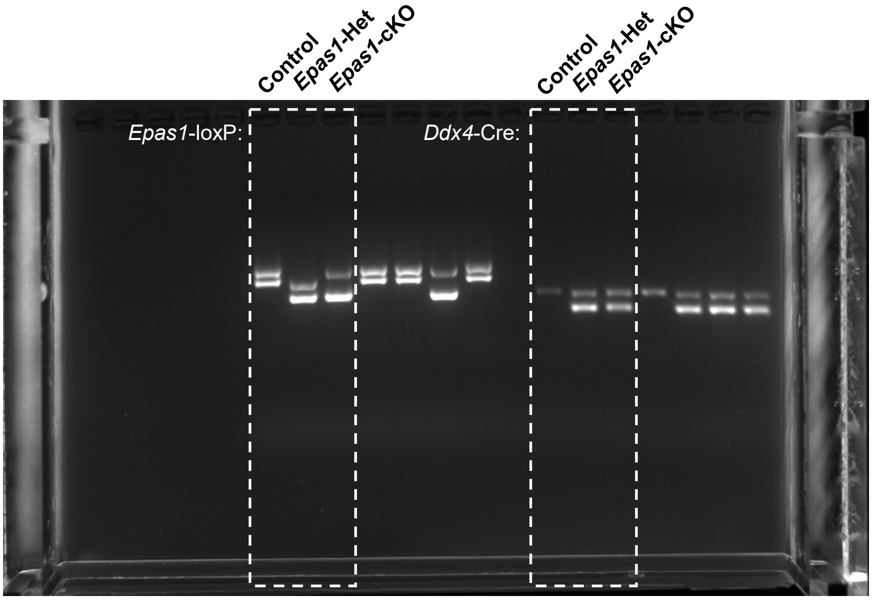

B

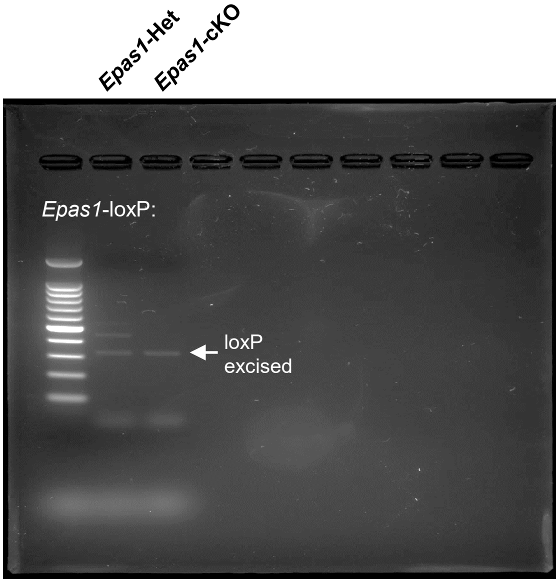

Figure S1 *(relates to Figure 2):* **(A)** Uncropped version of genotyping gel presented in Fig. 2A **(B)** PCR analysis of oocytes collected from heterozygous and Epas1-cKO females, demonstrating efficient excision of the loxP allele in the cKO such that it is no longer detectable.

SUPPLEMENTAL FIGURE 2

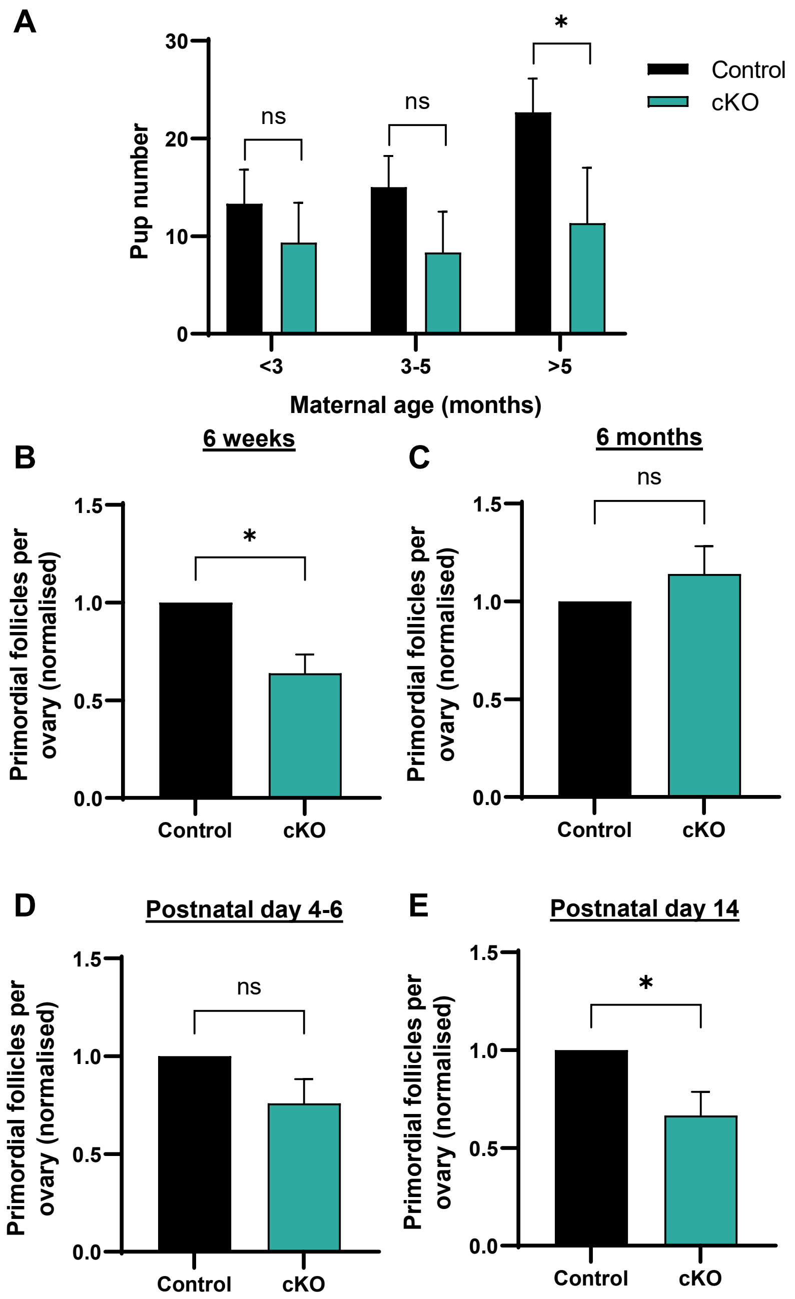

Figure S2 *(relates to Figure 4 and 5).* **(A)** Pups produced by control or *Epas1*-cKO females (paired with control males) as a function of maternal age. Histogram shows mean ± S.E.M. with values normalised to the control, n=3 biological replicates, * indicates statistical significance at $p<0.05$. **(B-E)** Primordial follicle counts for control and *Epas1*-cKO ovaries from adult females at 6 weeks (B) and 6 months (C) of age, and neonates / pre-pubescent females at postnatal day 4-6 (D) and 14 (E), respectively. Data here are based on estimates of total primordial follicle reserve using a serial sectioning approach. Histogram shows mean ± S.E.M. with values normalised to the control, n=4 biological replicates, * indicates statistical significance at $p<0.05$.

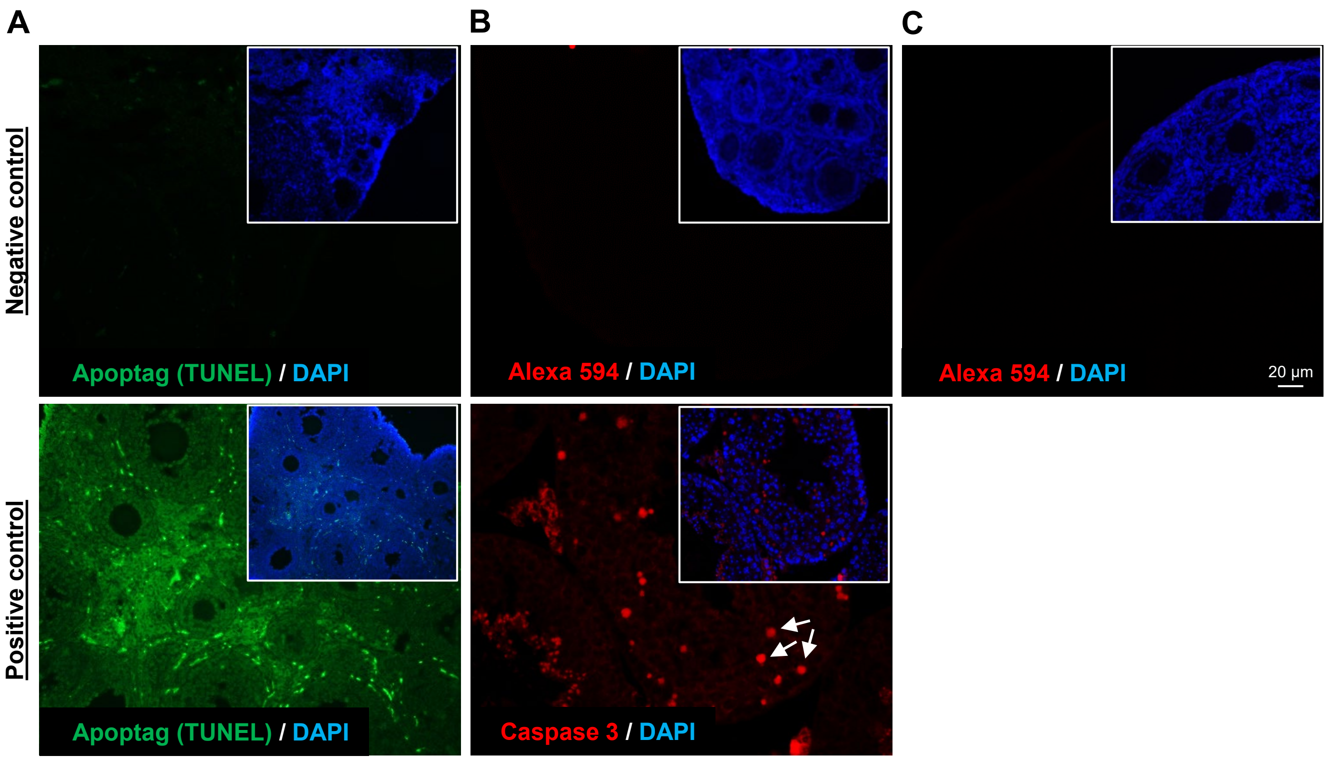

Figure S3 *(relates to Figure 6).* **(A)** Positive and negative control images accompanying TUNEL (Apoptag) staining in control and *Epas1*-cKO ovaries in Fig. 6A. Negative control had Tdt enzyme omitted, and positive control was treated with DNase to induce DNA fragmentation. Scale bar = 20 µm. **(B)** Cleaved Caspase 3 positive and negative control images accompanying images in Fig. 6A. Negative control had primary antibody omitted, positive control was an adult mouse testis section harbouring apoptotic cells (white arrows). Scale bar = 20 µm. **(C)** Negative control image in which primary antibody was omitted, accompanying autophagy images presented in Fig. 6B. Scale bar = 20 µm.

Table S1: Primer sequences for genotyping

GENE	PRIMER	PRODUCT SIZE
Ddx4-Cre	Internal positive control:	324 bp
(genotyping)	Fwd: 5’ CTA GGC CAC AGA ATT GAA AGA TCT 3’	
	Rev: 5’ GTA GGT GGA AAT TCT AGC ATC ATC C 3’	
	Cre:	240 bp
	Fwd: 5’ CAC GTG CAG CCG TTT AAG CCG CGT 3’	
	Rev: 5’ TTC CCA TTC TAA ACA ACA CCC TGA A 3’	
Epas1-fl	Fwd: 5’ CAG GCA GTA TGC CTG GCT AAT TCC AGT T 3’	Floxed allele: 444 bp
(multiplex PCR	Rev 1: 5’ CTT CTT CCA TCA TCT GGG ATC TGG GAC T 3’	Wild type allele: 410 bp
genotyping)	Rev 2: 5’ GCT AAC ACT GTA CTG TCT GAA AGA GTA GC 3’	KO allele: 340bp
